# Mechanisms of Winner-Take-All and Group Selection in Neuronal Spiking Networks

**DOI:** 10.3389/fncom.2017.00020

**Published:** 2017-04-21

**Authors:** Yanqing Chen

**Affiliations:** The Neurosciences InstituteLa Jolla, CA, USA

**Keywords:** neuronal spiking network, phase transition, learning and memory, Winner-take-all (WTA), neural computation, Robotics

## Abstract

A major function of central nervous systems is to discriminate different categories or types of sensory input. Neuronal networks accomplish such tasks by learning different sensory maps at several stages of neural hierarchy, such that different neurons fire selectively to reflect different internal or external patterns and states. The exact mechanisms of such map formation processes in the brain are not completely understood. Here we study the mechanism by which a simple recurrent/reentrant neuronal network accomplish group selection and discrimination to different inputs in order to generate sensory maps. We describe the conditions and mechanism of transition from a rhythmic epileptic state (in which all neurons fire synchronized and indiscriminately to any input) to a winner-take-all state in which only a subset of neurons fire for a specific input. We prove an analytic condition under which a stable bump solution and a winner-take-all state can emerge from the local recurrent excitation-inhibition interactions in a three-layer spiking network with distinct excitatory and inhibitory populations, and demonstrate the importance of surround inhibitory connection topology on the stability of dynamic patterns in spiking neural network.

## 1. Introduction

Facing with vast amount of multi-sensory information, Central Nervous System (CNS) seems to process only a small subset of those inputs at any given time, no matter whether they come from external or internal sources. How brain selectively processes such large number of inputs and maintains a unified perception remains a mystery. At the level of neuronal networks, a network in which all neurons respond the same to all stimuli would convey no information about the stimulus. In order to be useful, neurons must come to respond differentially to variety of incoming signals. Many neural models and theories have been proposed to account for such ability. Winner-Take-All (WTA) network is one of such proposed mechanisms for developing feature selectivity through competition in simple recurrent networks, and it has received much attention on both theoretical and experimental grounds. The primary theoretical justification is the ability of such networks to explain how the maps, which are ubiquitous in the cerebral cortex, can arise (Kohonen, 1982; Goodhill, 2007). WTA networks can also explain how a network can come to make useful distinctions between its inputs. WTA networks coupled with synaptic learning rules and homoestatic plasticity can explain how this takes place in a self-organized fashion from an initially undifferentiated state. Finally, WTA models are often employed at the behavioral level in theoretical models of higher-level cognitive phenomenon such as action-selection, attention (Itti and Koch, 2001; Walther and Koch, 2001) and decision making (Wang, 2002; Furman and Wang, 2008).

Another mechanism proposed for feature selectivity is the phenomenon of spatially localized bumps in neuronal networks (Somers et al., 1995; Laing and Chow, 2001; Wei et al., 2012). If we view multiple neurons within a bump as mulitiple-winners of excitatory and inhibitory competition, bump activity in spiking networks can be treated as a soft WTA or k-Winner-Take-All phenomenon (see Maass, 2000 for their definition). In this paper we use Winner-Take-All (WTA) and “bump activity” inter-changeably to describe the same stable group activity that arises from inter-connected excitatory-inhibitory neuronal networks. On a more general level, both bump activity and WTA phenomenon can be viewed as a type of pattern formation process in networks of excitatory and inhibitory neurons (for example, patterns of stable grid in Wilson and Cowan, 1973; Ermentrout and Cowan, 1979), and an example of activity dependent neuronal group selection process (Edelman, 1987).

Population rate-based WTA models have been extensively studied and are well understood (Dayan and Abbot, 2001). But, the connections between rate models to the real biological neural systems are not direct, because they are different from the real nervous systems whose neurons are spiking. So it is necessary to study the networks of spiking neurons, such that the biological interpretation of spike models can be more directly linked to real nervous systems. Modeling and understanding spiking networks is not simple because spiking neurons are highly nonlinear and their action potentials are discrete. As a result, it is always more difficult to obtain analytical solutions for spiking firing properties than rate models.

Analysis has shown conductance-based spiking models can be approximated by simple rate models under certain conditions (such as in an asynchronous state in Shriki et al., 2003). This approach has been applied to the study of hyper-column in a spiking model of visual cortex (Shriki et al., 2003). The orientation selectivity in their study, is modeled as the appearance of a unimodal “bump”-like spiking activity in a ring-connected spiking network, similar to an earlier study (Laing and Chow, 2001). Both approaches applied approximations from the rate models and used Fourier analysis to calculate the conditions for the appearances of bump activity. Recent work specifically studied recurrent spiking WTA networks, which are closer to real biological systems than previous rate models (Rutishauser and Douglas, 2009; Rutishauser et al., 2011). Even though these newer network models can receive spike input and generate spike output, their network structures are still very simplified. For example, excitatory and inhibitory neurons are modeled into one single population (Laing and Chow, 2001), and inhibitory population are reduced into one unit (Rutishauser et al., 2011), or removed altogether and modeled as direct inhibitory connections among excitatory neurons (Oster et al., 2009).

In a recent report we presented a robust and more biologically-realistic WTA network structure with distinct excitatory and inhibitory populations with arbitrary number of units (Chen et al., 2013). This WTA network has been implemented into a robot that accomplished a sequence learning and mental rotation task (McKinstry et al., 2016). In our spiking models each neuron type has very detailed biological parameters to model different neuronal transmitters and receptor types similar to previous work (Izhikevich and Edelman, 2008). We showed that surround inhibition and longer time constants from NMDA and GABAb conductances are sufficient to achieve stable “bump” spiking activity in a selected winner neuronal group while all the other neurons are inhibited and quiet. However, detailed biological properties, such as STSP (short-term synaptic plasticity), NMDA voltage gating etc., prevented a formal analytical analysis of the whole model. Also, it is not clear any of those biological details or a specific type of synaptic connections are crucial for the emergence of bump activity.

To identify the most important mechanistic factors for the spiking WTA networks, here we study a simplified spiking network after some biological details are removed. For example, based upon what we have noticed previously, turning off STSP, NMDA voltage-gating and excitatory-to-excitatory connections does not change the overall properties of WTA phenomenon. On the other hand, we preserve some important biological features such as the four different synaptic connections and conductance types (AMPA, NMDA, GABAa, and GABAb), because we found that these four individual conductance types contribute to different aspects of the “bump" stability. By examining functions of these individual conductances and the topologies of excitatory-inhibitory connectivity, we provide a detailed analysis of the conditions on which a stable bump activity can emerge from this recurrent spiking network. Our analysis thus provide a mechanistic analysis on how a neuronal group selection process can occur in an activity dependent manner in neural systems.

## 2. Methods

### 2.1. Network structure

Here we analyze a basic 3-layer spiking neuronal network with different neuron types with realistic biological parameters. The first layer of excitatory neurons (IN – input cells) takes input signals (e.g., arbitrary analog patterns) and translates them into spiking activity. The input signal we considere here in this paper is a type of unstructured random currents evenly distributed within a certain range and injected into the 100 input neurons (IN). IN cells are randomly connected to the next excitatory layer (E) with initial weights evenly distributed between 0 and a maximal value. The random input currents and random connections to the excitatory layer we analyzed here provide a baseline condition in which we test how the recurrent/reentrant connectivity between excitatory and inhibitory neurons by themselves can accomplish winner-take-all competition to random but unstructured input patterns (without obvious firing-rate differences among input neurons) and without synaptic modifications. The successful WTA network structure then can be trained to discriminate more complex and structured patterns through spike-timing dependent learning rules such as STDP. Such learning process will modify the synapses between these two excitatory types so that a selected E and I neurons (the WTA group) will respond to preferred input patterns more quickly for practical applications. We have demonstrated these in previous reports (Chen et al., 2013; McKinstry et al., 2016) where the same WTA network structures were implemented in a humanoid robot to process real world complex visual inputs, to learn visual-motor association and sequencing, and to accomplish a “mental rotation" and delayed-match-to-sample task.

We also implemented the above network using adaptive exponential spiking models and obtained similar results. For simplicity the analysis below uses the Izhikevich model (Izhikevich and Edelman, 2008), and excitatory (E) and inhibitory (I) neurons use the same parameters in the following equation:

(1a) $$\begin{array}{ll} C\dot{v} & =k\left(v-v_{r}\right)\left(v-v_{t}\right)-u-I_{syn} \end{array}$$

(1b) $$\begin{array}{ll} \dot{u} & =a\left\{b\left(v-v_{r}\right)-u\right\} \end{array}$$

Parameters in these equations are the same as explained before (Izhikevich and Edelman, 2008). That is, *v* is the membrane voltage in millivolts (*mV*), *C* is the membrane capacitance, *v*_*r*_ is the neuron's resting potential, *v*_*t*_ refers to its threshold potential, *u* represents the recovery variable defined as the difference of all inward and outward voltage-gate currents. *I*_*syn*_ is the synaptic current (in *pA*) originated from spike input from other neurons. *a* and *b* are different constants. When the membrane voltage reaches a threshold, i.e., *v* > *v*_*peak*_, the model is said to generate a spike, and two variables in Equations (1a, 1b) are reset according to *v* ← *c* and *u* ← *u* + *d* while *c* and *d* are parameters for different cell type.

We use a simplified synaptic current form with four basic conductances from *AMPA*, *NMDA*, *GABA*_*A*_, and *GABA*_*B*_ channels. For simplification, voltage-gating of NMDA channel is reduced to a constant factor. This is done through calculating an average number for the voltage-gating term for the NMDA conductance (i.e., [(*v* + 80)/60]^2^/[1 + ((*v* + 80)/60)^2^] on Page 11 of the Supplementary Information in (Izhikevich and Edelman, 2008)) for the normal range of voltages: *v* = [−60, 60], and the result is equivalent to a voltage-independent NMDA channel with smaller gain factor than AMPA channels (see Appendix for the individual conductance gain factors we used). So synaptic current *I*_*syn*_ is composed of four different current types originated from those four conductances multiplied with the voltage differences between their individual reversal potentials:

(2) $$\begin{array}{lll} I_{syn}\; & = & \;g_{AMPA}\left(v-0\right)\;+\;g_{NMDA}\left(v-0\right)\;+\;g_{GABA_{A}}\left(v+70\right)\\ \;+\;g_{GABA_{B}}\left(v+90\right). \end{array}$$

Shown in the above equation, reversal potentials of *AMPA* and *NMDA* channels are 0 and reversal potentials for *GABA*_*A*_ and *GABA*_*B*_ channels are −70 and −90*mV* respectively.

As described before, each conductance has exponential decay with different time constants in millisecond (ms):

(3) $$\begin{array}{l} \dot{g}=-g/\tau , \end{array}$$

while τ = 5, 150, 6, and 150 for the *AMPA*, *NMDA*, *GABA*_*A*_, and *GABA*_*B*_ channels respectively.

**Figure 1:**
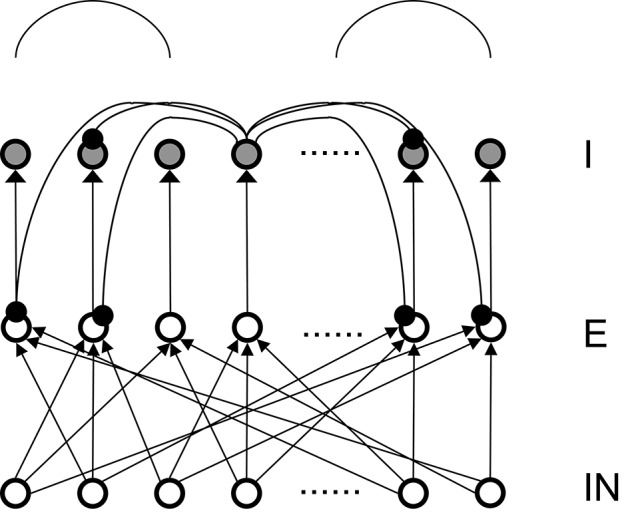
**Structure of the basic 3-layer spiking network and a schematic plot of the “surround inhibition” connectivity that supports winner-take-all phenomenon**. IN – thalamo-cortical input neurons, E – Excitatory pyramidal neurons, I – Inhibitory neurons. We chose 100 input (IN) cells, 400 E cells, and 400 I cells for total of 900 neurons in the analysis model presented here. Input layer to excitatory layer (IN to E) are all-to-all random connected, excitatory to inhibitory layers are narrow and were simplified into one-to-one connection in our analysis. Inhibitory connections are surround type, that is, I cells do not inhibit its nearest neighboring I and E cells, but only distant surrounding neurons. This connectivity is implemented as two cosine peaks with a flat gap (zero value connectivity) in between. We call this specific network connectivity as surround inhibition type for the one dimensional case and it is a simplified version of the two-dimensional Central-Annual-Surround (CAS) type of topology we described before (Chen et al., 2013).

To simplify the analysis, there are equal numbers (400 in all the subsequent analysis) of excitatory (E) and inhibitory (I) neurons in our basic network model in Figure 1, although their numbers can be in any ratio. In fact, in our previous published full models (Chen et al., 2013; McKinstry et al., 2016) the ratio of E and I neurons were set at 4:1 to more closely resemble the real cortex. We also explored different types of connection topologies in the connections from excitatory to inhibitory neurons (E to I), the reentrant inhibition from basket cells to pyramidal neurons (I to E) and the inhibitory connections within basket cells themselves (I to I). In our study, Inhibitory to Excitatory and Inhibitory to Inhibitory connections are kept the same topological type and total weights are kept equal. Throughout the simulation the total connection weights to each neuron are normalized to be a constant for each connection type. The total weights for each connection type (E to I and I to E) are two parameters we explored systematically. As a first step, we firstly only consider one type of inhibitory conductance (*GABA*_*A*_) to obtain analytical solutions for the conditions of Winner-Take-All state. *GABA*_*B*_ conductances are added after an analytical solution is found, a comparison of the transition plots can be found in the Appendix.

## 3. Results

**Figure 2:**
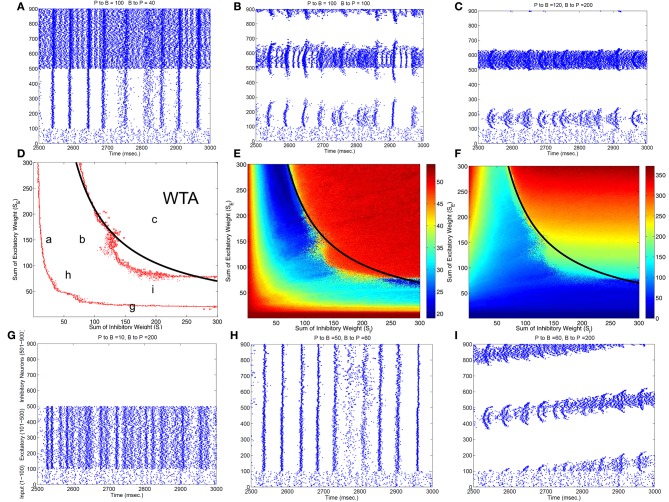
**Classification of different dynamic spiking patterns in the surround inhibition network we defined in Figure 1 and the phase diagram for the transition between different firing states**. **(A–C,G–I)** Are raster plots which show all spikes within a half second interval for each neuron in the network. **(E,F)** are maximal firing rates of excitatory and inhibitory neurons in the 2-d parameter space (total excitatory weight in y-axis and total inhibitory weight in the x-axis). Red Points in **(D)** are transition curves constructed from **(E)** where maximal firing rate of excitatory neurons changed from below to above 40 Hz. **(A–C,G–I)** Are example spiking patterns under their specific parameter combinations which are marked on the 2-d plot in **(D)**. Subplot **(C)** represents a winner-take-all (WTA) state where only a small group of excitatory and its corresponding inhibitory neurons fire persistently while others are silent. Black curves on the middle row–**(D,E,F)** are the same analytic transition condition for the WTA region based upon the analysis described in the Appendix (Equation A.14). Basically it is a curve where *S*_*E*_ · *S*_*I*_ equals to a constant defined by Equation (A.14), and it delineates the Winner-Take-All region (i.e., max firing rate large than 40 Hz for only a selected group of excitatory neurons) in the parameter space very well for both the excitatory and inhibitory neurons.

To classify different types of spike dynamics for the surround inhibition network in Figure 1, for each neuron, we record the number of spikes between 2 and 3 s after the simulation had reached steady state without synaptic plasticity (STDP off). We then characterize the behavior of the network by the maximum number of spikes generated by any excitatory neuron. Figure 2E plots this maximal firing rate for every combination of the E to I weights vs. the I to E weights. The analysis is repeated and plotted in Figure 2F for the inhibitory population.

Figure 2 shows different types of dynamic firing patterns in the 2-dimensional parameter space. When only one type of connection weight (excitatory or inhibitory) is high but the other weight is low, either excitatory or inhibitory neurons are in a quasi-random/rhythmic state in which one group of neurons fires in high Gamma frequency range (>40 Hz, see Figures 2A,G). When both connection weights are relatively high (see Figure 2C), both excitatory and inhibitory neurons have high maximal firing rates where excitatory neurons have a maximal firing rate larger than 35 Hz and inhibitory neurons have a maximal firing rate of larger than 100 Hz. If we look at the corresponding spike raster plot in Figure 2C, only a subset of excitatory and inhibitory neurons maintain such high firing rates while majority of other neurons are silent. We call this Winner-Take-All (WTA) state in which only a small subset of neuronal groups persistently fire high frequency and keep the rest of neurons from firing using surround inhibition. The region of the parameter space with such WTA states is delineated by the right curve in Figure 2D where the maximal firing rate of excitatory neurons increased to greater than 35 Hz from lower firing rates in the middle region (from the blue area in Figure 2E transition to the red area on the top right), and roughly corresponds to a similar increase of maximal firing rate to above 100 Hz for inhibitory neurons in Figure 2F.

Subplots Figures 2A,B,H,I all belong to an intermediate region in the parameter space in Figure 2B between two curves where maximal firing rates for both excitatory and inhibitory neurons are relatively low. Within this parameter range, excitatory and inhibitory neurons are either quasi-synchronized (Figure 2A) or precisely synchronized and firing rhythmically (Figure 2H), or exhibit moving bump activity (Figure 2I) or as combinations of rhythmic and moving bump activity. In all these cases, single excitatory neuron cannot maintain a stable high gamma frequency spiking activity unless connection weights are changed, moving to the WTA region on the top-right of the second curve in Figure 2D. Figure 2D thus provides a phase diagram for the neuronal network defined in Figure 1.

Notice that this maximal firing rate is not the neuron's instantaneous firing rate, but is the total number of spikes within a 1 s window. This definition is useful to discriminate a stable high firing rate neuron vs. a neuron firing a short burst less than 1 s and then becoming quiet (especially for stable vs. traveling activity, see Figure 2C vs. Figure 2I).

**Figure 3:**
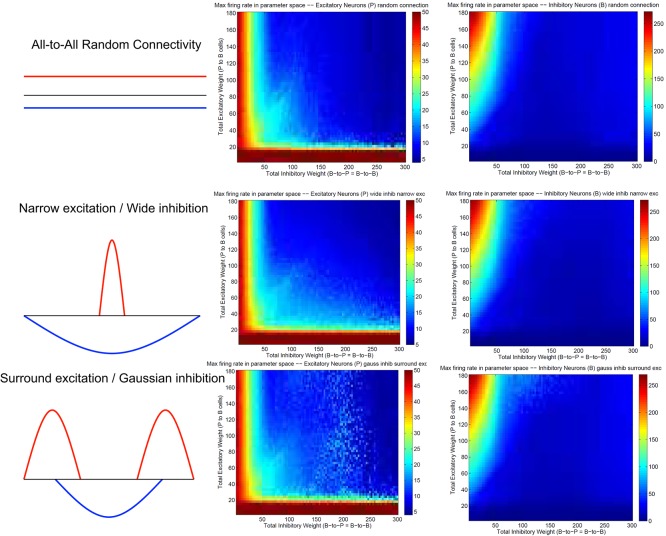
**Spiking dynamics under different connection types other than the surround inhibition type defined in Figure 1**. Each row represents a connectivity type and the middle and right columns are maximal firing rate of excitatory and inhibitory neurons under specific connectivity. These plots were calculated the same way as Figures 2E,F. Notice that all three connectivity types here do not support a winner-take-all (WTA) region in the parameter space (no red region in the upper-right corner). It exists in Figures 2E,F as a red region representing a high individual maximal firing rate state when both excitatory and inhibitory weights are relatively high, but it is always absent here on the top right of the 2-d parameter space.

Figure 3 summarizes patterns of spike dynamics with different connection topologies. Compared to the surround inhibition type analyzed above, all the other connection types do not support a Winner-Take-All state manifested as stable bump activity shown in Figure 2C. This is because under those connection types, excitatory and inhibitory neurons cannot maintain high maximal firing rates when both excitatory and inhibitory weights are high and did not have a red area on the upper-right region shown in Figures 2E,F. The most common firing patterns for those connection types are quasi-rhythmic firings in the 10–20 Hz range for excitatory neurons resembling an epileptic state while some short burst of unstable bump activity in inhibitory neurons. Our results suggest that, among different types of connectivity topologies we analyzed, only surround inhibition can generate a stable bump spiking activity and maintain a WTA state.

### 3.1. Mechanism of winner-take-all neuronal group selection and emergence of bump activity

Our above analysis suggests that surround inhibitory topology supports emergence of bump activity. To explore the mechanism of WTA and which neuronal properties are essential for such behavior, we applied the same analysis as in Figure 2 to neuronal network in Figure 1 when Short-Term-Synaptic-Plasticity (STSP) or NMDA voltage-gating is on, or change excitatory and inhibitory neurons' parameters to different type. In all cases, a similar WTA region was found for every conditions, even though the transition curves that delineate the emergence of stable bump activity are shifted to different positions in the parameter space (see results in Chen et al., 2013). We also analyzed the same neuronal network with a different set of individual spiking models, i.e., the adaptive exponential models and found the similar WTA region as long as the topology of the inhibitory connections are surround type. These analyses suggest that detailed neuronal properties such as exact models of the spiking neuron, STSP or NMDA gating etc., are likely not fundamental for the existence of stable bump activity, but the type of connectivity topology (i.e., surround inhibition) is more important for such behavior.

**Figure 4:**
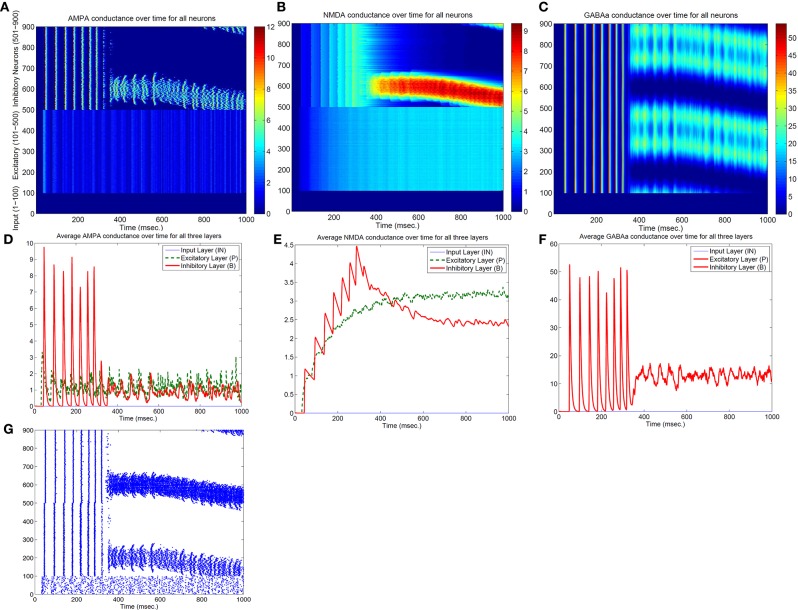
**Transition from a synchronized rhythmic firing state to a winner-take-all bump spiking activity–(G)**. In this simplified surround inhibition network, short-term synaptic plasticity (STSP) and NMDA gating effects are removed, GABAb conductance is set to be zero and individual excitatory and inhibitory spiking models are changed to have the same biological parameter set. First row shows the 1-s spatial-temporal evolutions of individual conductance for AMPA–**(A)**, NMDA–**(B)**, and GABAa–**(C)**. Second row **(D–F)** show the averaged conductance over time for excitatory and inhibitory cells separately. Notice that bump activity in AMPA and NMDA conductance appear in inhibitory neurons first (see **A,B**), while both conductances are spatially uniform even after spiking bump activity emerged after about 400 ms. The fact that spatial uniformity is destroyed in GABAa conductance first in **(C)** suggests that inhibitory neurons might show transition into winner-take-all state early and then bias the transition in excitatory cells.

Both the Izhikevich neural model and the adaptive exponential model we used are conductance based with models of inhibitory and excitatory currents of different time scales. So we suspect that different time constants of NMDA, AMPA, GABAa, and GABAb channel conductance might play some role for the emergence of bump activity. To demonstrate this, Figure 4 shows the time evolutions of AMPA, NMDA, and GABAa currents along with the spiking activity in the simplified network in Figure 1 starting from a zero conductance initial condition. It demonstrates the detailed transition from a rhythmic synchronized firing state into a stable bump activity. Looking at detailed dynamic changes of the individual excitatory and inhibitory currents should shed light on how the transition is occurred.

From Figure 4 we can see, differences in time constants will determine how fast a specific channel conductance returns back to zero after a burst of spiking activity. For excitatory neurons specifically, because of its short time constants (6 ms), AMPA current fluctuates around a similar level with large variances. NMDA currents, on the other hand, are accumulating to higher levels because of longer time constant (150 ms) even though both currents are generated by the same spiking input from the input neurons. Similar phenomenon can be seen for inhibitory neurons. When those neurons fire rhythmically before about 300 millisecond, AMPA conductance jumps to high level (from 7 to 9 nS) after each spike then drops down to zero very fast (red curve in Figure 4D), while NMDA conductance only drops a small amount each cycle and overall level still increases to much higher value (red curve in Figure 4E). Initially inhibitory neurons fire after excitatory neurons in each rhythmic cycle and they synchronize to each other with a time delay. If excitatory to inhibitory weights (E to I) are larger than a certain value, such that NMDA currents for inhibitory neurons increase faster than excitatory neurons (Figure 4E), the delay between inhibitory and excitatory neurons diminishes and GABAa currents become effective within the same cycle to inhibit other neurons. As a result some inhibitory and excitatory neurons stop firing in the rhythmic cycle, eventually lead to a winner group that persistently fires and shuts off their surrounding neighbors. Notice that in the simplified model in Figure 4, GABAb (with longer time constant of 150 ms) currents are omitted and set to zero, which lead to a moving bump activity for this specific parameter set. If GABAb conductance is restored to the original level as in the full model, bump activity becomes stable. It implies that time constant of GABAa and GABAb channel conductance is related to the stability of the bump activity.

As a summary, we think the combinations of long and short time constants from excitatory and inhibitory conductance plus the surround inhibitory connectivity support a mechanism for emergence of bump activity and winner-take-all phenomenon in this basic spiking neuronal network. This neuronal group selection mechanism provides a basis for modeling learning and map-formation process for sensory motor integration and other higher cognitive processes.

## 4. Analytical analysis of the transition curve for WTA phenomenon

### 4.1. Differentiation in inhibitory conductances lead to spiking activity pattern transition and neuronal group selection

**Figure 5:**
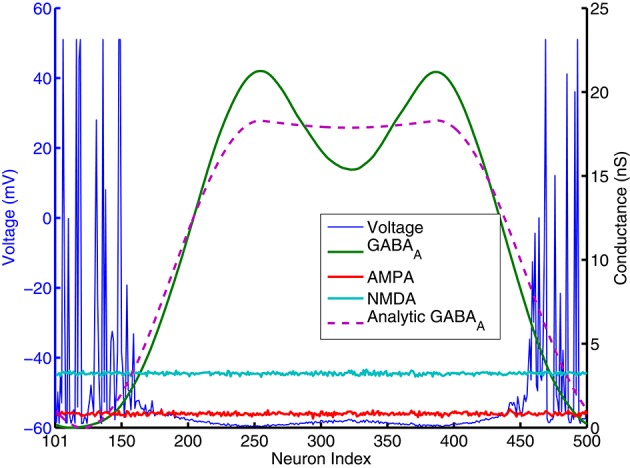
***AMPA, NMDA, GABA*****_*A*_ conductances and voltages for all excitatory neurons (cell index 101–500 in Figure 1) at one specific time point (*t* = 980 ms. in Figure 4)**. AMPA and NMDA conductances are basically flat across all excitatory neurons here, while *GABA*_*A*_ conductances are close to 0 for neurons around number 101 and 500, and reach high values else where. Pink dashed lines are analytic GABA conductances from Equation (A.6) in the Appendix and derived from surround inhibition topology, and is a good match for the actual numerical simulation. Notice that excitatory neurons fire spikes and have above threshold voltage values (blue lines) only within neighborhood of neurons having *GABA*_*A*_ conductances close to 0 and below a certain value.

To identify the mechanism of bump activity in spiking networks, based upon the transition plots shown in Figure 4, we look at voltages, conductances of all 400 excitatory neurons at one specific time point (*t* = 980 ms. in Figure 4). Figure 5 shows AMPA, NMDA, and GABAa conductances along with voltages for all those excitatory neurons at this specific time point. From the network structure defined in Figure 1, we know that the excitatory conductances (AMPA and NMDA) are determined by excitatory synapses from the input layer (because we omitted self-excitation), where inhibitory conductances (GABAa and GABAb) are only determined (triggered) by spikes from inhibitory neurons onto those excitatory neurons. Because of the uniform random connection from input layer, AMPA and NMDA conductances are around the same level and undifferentiated for all excitatory neurons. From Figure 5, voltages are above threshold and neurons fire only at locations where GABAa conductances below a certain value. So in order for a bump to emerge and a subgroup of neurons selected to be active, GABAa or GABAb conductances have to be differentiated, i.e., they have to be small for some neurons and remain high for all other neurons. We suspect this condition can only be met by surround type of inhibitory connectivity. It is obvious that lowest GABA conductances lead to highest firing rate for excitatory neurons. Since we have local feedforward excitation to inhibitory neurons in our network, the bump area in inhibitory neurons with highest firing rate should also have lowest inhibitory conductances. This difference in GABA conductances is true for both excitatory and inhibitory neurons because GABA conductances are determined by the same inhibitory spikes. This suggests that in order for a bump to emerge, local inhibition to the nearest neighbors should be lower than inhibition to neurons outside of the bump. Notice the three other inhibition topology in Figure 3 all have peak (or flat) inhibition locally, so even if a neuronal group emerge with highest firing rate, the strong local inhibition will force their firing rates to decrease, and let the other sub-threshold neurons to fire. So this is likely the reason why we did not obtain stable bump activity using those inhibition connectivities. On the other hand, surround inhibition type defined in Figure 1 might be the most simple form of inhibition topology that could let a bump emerge and stabilize. Below analysis will further prove this point.

In contrast with models using negative weights to represent inhibitory connections, our spiking models' synaptic weights and excitatory/inhibitory conductances are all positive (which obviously is more biologically realistic). From Equation (2) we can see, it is only because of the differences in reversal potentials between excitatory and inhibitory channels, the current generated by excitatory and inhibitory conductances could have different signs (excitatory current coming into the neuron and inhibitory current coming out of neuron). In order for a neuron to fire, synaptic current has to be below a threshold *I*_*syn*_ < −*I*_*th*_ where *I*_*th*_ is about 100pA (*I*_*syn*_ has to be negative for a neuron to fire because it was defined as an outward current in Equation 1a). Firing threshold *I*_*th*_ can be found from calculating F-I curve for the specific spiking neuron model we used. Figure A.3 (in the Appendix) plots the firing rates vs. amount of injected current (equivalent to −*I*_*syn*_) for the Izhikevich neuron model result from numerical simulations. It shows that neurons start to fire when absolute value of the injected current is above 100 pA and then increase their firing rate approximately linearly until above 100 Hz. we can use this information to simplify the spiking activity into a rate model. As indicated on the last paragraph, AMPA and NMDA conductances are approximately uniform for excitatory neurons and they can not contribute to the differences in firing rates, so in order for the excitatory population to fire differentially, the difference between highest and lowest GABA conductances for individual neurons has to be larger than a certain value. This value can be estimated using Equation (2). If *min*(*g*_*GAB*_*A*__*A*__) is 0, for a resting potential of *v*_*r*_ = −60*mV*, *GABA*_*A*_ conductance has to be larger than the following value so injected synaptic current −*I*_*syn*_ will be below the firing threshold *I*_*th*_:

(4) $$\begin{array}{l} g_{GABA_{A}}>\left(-I_{th}+60*\left(g_{AMPA,E}+g_{NMDA,E}\right)\right)/10. \end{array}$$

In Figure 5, *g*_*AMPA,E*_ + *g*_*NMDA,E*_ for excitatory neurons is around 4nS (Appendix will show how this value can be estimated analytically), so *g*_*GAB*_*A*__*A*__ has to be larger than 14 nS to keep sub-threshold neurons from firing. This number is consistent with the result plotted on Figure 4, 5 that neurons fire and form a bump area where GABA conductances are below 14 nS and areas with GABA conductance larger than 14 nS are completely quiet.

If we consider both the *GABA*_*A*_ and *GABA*_*B*_ conductances based upon Equation (2) and using the same idea as above, conditions for inhibitory conductances will be the following for the winner-take-all state:

(5) $$\begin{array}{l} 10\cdot g_{GABA_{A}}+30\cdot g_{GABA_{B}}>\left(-I_{th}+60*\left(g_{AMPA,E}+g_{NMDA,E}\right)\right). \end{array}$$

Equations (4, 5) can be used further to identify the exact condition for the WTA state and to locate the transition curve in Figure 2. Using two cosine bumps as surround inhibitory connection weights, Appendix gives the analytic form of GABA distribution of a bump solution for neurons connected one dimensionally and uses it to obtain analytic conditions for the WTA state in the parameter space (see Equations A.13, A.14). Such analytic conditions are expressed as formulas combining single neuron property and the conductance parameters (such as time constants, gain factors for different inhibitory and excitatory conductances). Based upon these formulas we can locate the Winner-Take-All and bump activity in the parameter space fairly precisely (see black curves in Figure 2 and the white curve in Figure A.4 in the Appendix), thus provide a mechanistic explanation for the emergence of winner-take-all state and stationary bump activity in this 3-layer spiking network we analyzed here.

### 4.2. Origin of traveling wave and instability of bump activity – driven by AMPA conductances

**Figure 6:**
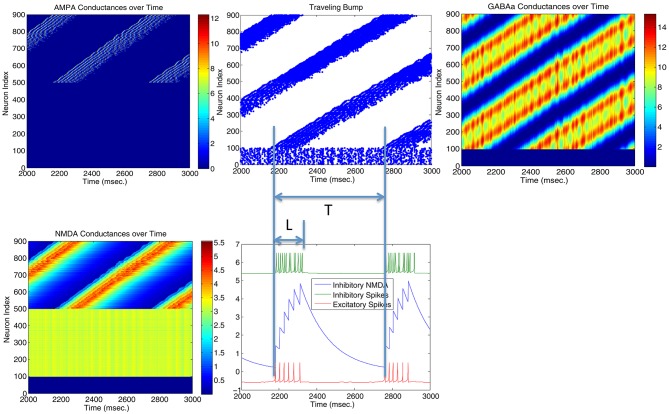
**Stability of the bump activity is determined by AMPA conductances**. Figures above show that spiking bump activity moves with a constant speed when AMPA conductances in excitatory neurons are blocked and set to 0 (Top left). The speed of the traveling “bump,” proportional to the length of firing interval for each neuron (L) and the period of the traveling wave (T), seems relate to the frequency of the inhibitory neurons (hence their AMPA conductances) and the delay time for the NMDA conductances when they reach maximal firing rate. When AMPA conductances are set to 0 in inhibitory neurons, bump activity stops moving and *T* → ∞.

Parameters in Figure 4 are located very near the transition curve in the parameter space (see Figure 2), so the bump is not spatially stable and moves across different neurons. To identify the origin of such instability, we selectively set AMPA gain of excitatory or inhibitory neurons to 0 in order to see their effects on the bump stability. This is equivalent to selectively block AMPA conductances in either excitatory or inhibitory neurons in real biological neural systems. We found that if AMPA conductances in inhibitory neurons are set to 0 but not in excitatory neurons, bumps become more or less stable. On the other hand, when AMPA conductances are blocked and set to 0 in excitatory neurons but not in inhibitory neurons, we can have a moving bump with a constant spatial speed (see Figure 6). In fact, we can estimate the moving speed of bumps based upon the parameters we defined in Figure 2. So we believe the source of the bump instability is from the AMPA conductances in inhibitory neurons. Previous studies have associated stabilities of bump activity with dynamic synapses (Fung et al., 2009). Notice that AMPA conductances have much shorter time constants than others thus more associated with faster synapses (similar to GABAa), so in this sense there might be a connection and some agreement between our observation on Figure 6 and dynamic synapses analyzed by Fung et al. (2009).

## 5. Conclusion and discussion

In this paper we derive global properties of spiking neuronal networks related to bump activity and Winner-Take-All state mainly through analysis of the dynamics of excitatory and inhibitory conductances. To achieve the analysis of collective behavior, individual spiking properties are approximated by its firing rate property such as the conductance/firing rate curve (Figure A.3 in the Appendix) or F-I curves. In this regard, detailed properties of individual spiking model might not be crucial for global activity such as the emergence of bump and WTA state. For example, if we use different parameter sets for excitatory and inhibitory neurons such as changing the inhibitory neurons to be basket cell type, we can still found the WTA region in the parameter space in Figure 2D, but the exact location of the transition curve is shifted to a different place because basket neurons have different conductance/firing rate curve and different *I*_*th*_, *g*_*th*_, *k* values in Equation (A.13). This could explain the transition curve in our full model with more detailed biological properties has the same functional form, but in different location in the parameter space (see Chen et al., 2013) because it included more detailed single neuronal spiking properties such as NMDA voltage gating and STSP etc. In fact, we used adaptive exponential spiking model to substitute the Izhikevich neuron models and obtained similar phase plot and transition curve for the bump activity and the WTA state.

We suggest that all conductance-based spiking models with distinct excitatory and inhibitory populations could have the similar collective Winner-Take-All behavior as analyzed here. Detailed spiking model properties such as F-I curve and firing threshold (*I*_*th*_ and *g*_*th*_) would determine the exact location of the transition curve in Figure 2. Global connectivity topology and different time constants (dynamics) of excitatory and inhibitory conductances are likely to be the determinant of system-wide spiking activity patterns.

### 5.1. Importance of the inhibitory topology

The most important feature of our winner-take-all network is its surround inhibition topology. The reason we chose two sine function peaks as surround inhibitory connection weights is just because of its mathematical convenience, since convolutions of sine/cosine functions are much easier to solve than other types of functions. In fact, connection topologies using Gaussian peaks or torus (for two-dimensional neuronal arrays) were used in our previous model (Chen et al., 2013) and similar stable WTA results were obtained. We believe using other type of function for inhibitory connection would also work, as long as there is a low inhibitory weight locally. Comparing four different connection topologies from Figures 2, 3, the reason why only surround-inhibition supports stable bump activities is because its maximal inhibitory connection weight is not to the nearest neighbors, but to slightly distant neurons. This gap of zero inhibitory weight can be very small (e.g., *w* down to 0 and equivalent to a no-self-inhibition case), and we can still find solutions for stable bump or bumps (in fact, *w* determines how many bumps can emerge and we will have a 2-bump solution when *w* is close to 0, see Appendix and Figures A.1, A.2). So as long as there is a local valley of inhibitory weight, stationary bumps could emerge because only decreasing inhibition could allow a bump to sustain.

Mechanistically it appears that the most important requirement for a bump solution is the stable differentiation in inhibitory (*GABA*_*A*_ or *GABA*_*B*_) conductance distributions across the neuronal population. That is, for some neuronal groups, GABA conductances should be low to allow bumps to emerge and for the other neurons, they need to be high enough to keep the rest of neuronal population from firing spikes. As long as this condition is met, more detailed biological properties such as local self-excitation, short-term synaptic plasticity (STSP), voltage-gating of NMDA channels etc. can be added to the model without destroying the overall bump stability.

### 5.2. Why traditional center-surround topology might not lead to stable bumps in models with distinct excitatory and inhibitory populations

Previous rate-based population models (Dayan and Abbot, 2001) had most often used center-surround type of connection topology as shown in the middle row of Figure 3 (Narrow excitation/Wide inhibition). Similarly, many spiking models with excitatory/inhibitory conductances on the same units used the same topology (Laing and Chow, 2001). By simple subtraction, narrow excitation and wide inhibition can lead to a “Mexican Hat” type of effective connectivity which supports winner-take-all in previous firing rate models. But, as we see from the analysis above, in biologically more realistic spiking models with distinct excitatory and inhibitory neuronal populations, multiple types of conductances cannot cancel each other easily because they are generated by precise spike timing and have different time constants. The “classical” center-surround topology can not guarantee a stable “Mexican Hat” type of net connectivity because sensitive spike timing differences between different neurons prevent easy subtraction of excitatory and inhibitory weights at every time point. In fact, as shown in Figure 4, the emergence of winner-take-all in spiking networks is a direct result of precise spike-timing–the coincide of excitatory and inhibitory population firing spikes lead to a sub-population of inhibitory neurons fire earlier than the rest of populations which then let them suppress and shut off the other neurons in the network (see Figure 4G). This is the reason that we believe a surround-type of inhibitory topology is essential for a stable spiking WTA network because it can support the emergence of a winner-group without shutting off themselves too early.

In summary, WTA network analyzed here demonstrates how variability and randomness in spiking time of individual neurons can lead to global pattern changes and phase transition in collective neuronal groups. Analytic solutions for the phase transition curve provided in this paper will help to increase our understandings of different functional roles of excitatory and inhibitory neural connections on the emergence and stability of firing patterns in the brain.

## Author contributions

YC developed the original idea of this paper, performed computational modeling and mathematical derivation for the analytical solution and wrote the paper.

### Conflict of interest statement

The author declares that the research was conducted in the absence of any commercial or financial relationships that could be construed as a potential conflict of interest.

## Appendix

The Appendix for this article can be found online at: http://journal.frontiersin.org/article/10.3389/fncom.2017.00020/full#supplementary-material
